# Bilateral pulmonary embolism in a patient with pulmonary tuberculosis: a rare association in Yaoundé, Cameroon

**DOI:** 10.11604/pamj.2014.17.262.4107

**Published:** 2014-04-10

**Authors:** Nkoke Clovis Ekukwe, Luchuo Engelbert Bain, Ahmadou Musa Jingi, Kotta Sylvia, Pierre Mintom, Alain Menanga

**Affiliations:** 1 Faculty of Medicine and Biomedical Sciences, Department of Internal Medicine, Yaoundé, Cameroon; 2 Military Hospital of Garoua, Cameroun; 3 Department of Internal Medicine, Yaoundé General Hospital, Yaoundé, Cameroon

**Keywords:** Pulmonary embolism, pulmonary tuberculosis, cardiomegaly, dyspnea

## Abstract

Pulmonary embolism is a complication of pulmonary tuberculosis that has received little emphasis in the literature. We describe a 52 year old male, with no risk factors for thromboembolic disease referred to our service for an in depth clinical review for cardiomegaly and dyspnea on exertion. Echocardiography and CT scans revealed dilated heart cavities and bilateral proximal pulmonary emboli respectively and a cavitation in the apical lobe of the right lung. Bronchial aspirate and culture revealed the presence of mycobacterium tuberculosis. There was no evidence of malignancy. Elsewhere, a clinical review and a lower limb ultrasound showed no evidence of deep venous thrombosis. Clinical course on anti - tuberculosis and anti - coagulant therapies was remarkably favorable. Clinicians need to be conscious of the risk of developing thromboembolic disease in patients treated for tuberculosis, in especially high prevalence settings like ours.

## Introduction

Tuberculosis is a major health problem in developing countries with a myriad of presentations and complications. Tuberculosis can lead to hypercoagulability, increased venous stasis, and endothelial dysfunction, thus increasing the susceptibility to venous thromboembolism (VTE). Our case highlights the occurrence of bilateral pulmonary embolism in a patient with pulmonary tuberculosis with no risk factors for thromboembolism, a significant but rare association posing a diagnostic dilemma which may have serious prognostic implications.

## Patient and observation

A 52-year old previously healthy man was referred from a pneumology hospital in the capital city of Cameroon, Yaoundé, for the evaluation of suspected heart failure. He consulted for shortness of breath on moderate exertion associated with a dry cough of two days duration. The cough was worse at night. There was no associated fever, no weight loss; no excessive sweating at night, no chest pain, no lower extremity swelling and no hemoptysis. He had no significant medical or family history of a cardiovascular or thromboembolic disease. His past history was only remarkable for exposure to a patient with tuberculosis ten years ago. He was not a current smoker and did not consume alcohol.

Physical examination revealed a well build up patient in no acute distress, a blood pressure of 110/70 mmHg, pulse rate of 70/min, respiratory rate of 19 cycles/min, temperature of 36.9°C. His weight was 71kg and BMI of 25.15kg/m ^2^. On the examination of the respiratory system, auscultation revealed clear breath sounds without wheezes nor crackles. On examination of the cardiovascular system, he had a regular heart beat without murmurs, no accentuation of the pulmonary component of the second heart sound, no jugular venous distension and no lower extremity edema.

**Figure 1:**
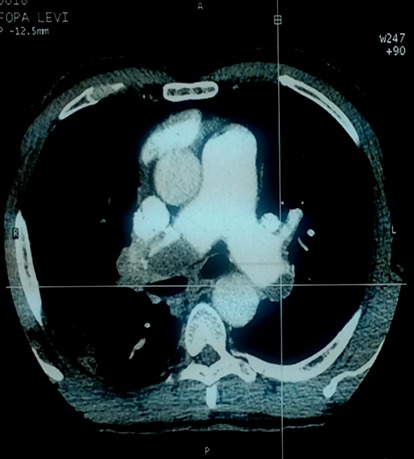
Thoracic CT scan showing thrombus in the right and left branches of the pulmonary arteries

**Figure 2:**
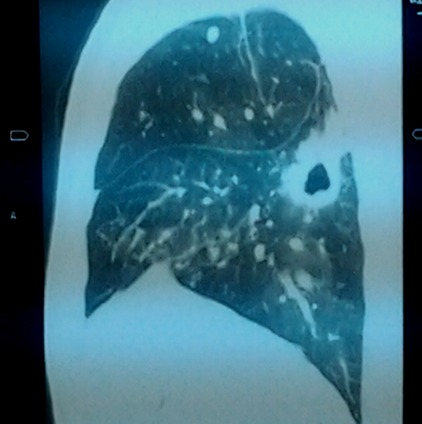
Thoracic CT scan showing cavitary lesion in upper lobe of the right lung

**Figure 3:**
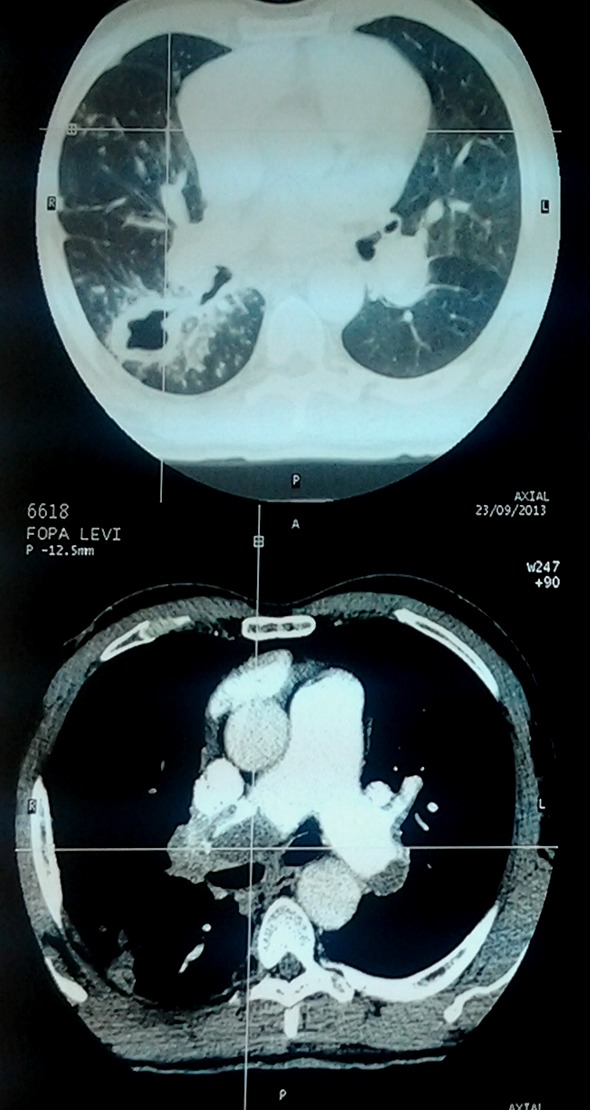
Thoracic CT scan showing cavitation in upper lobe of the right lung and bilateral pulmonary embolism

**Figure 4:**
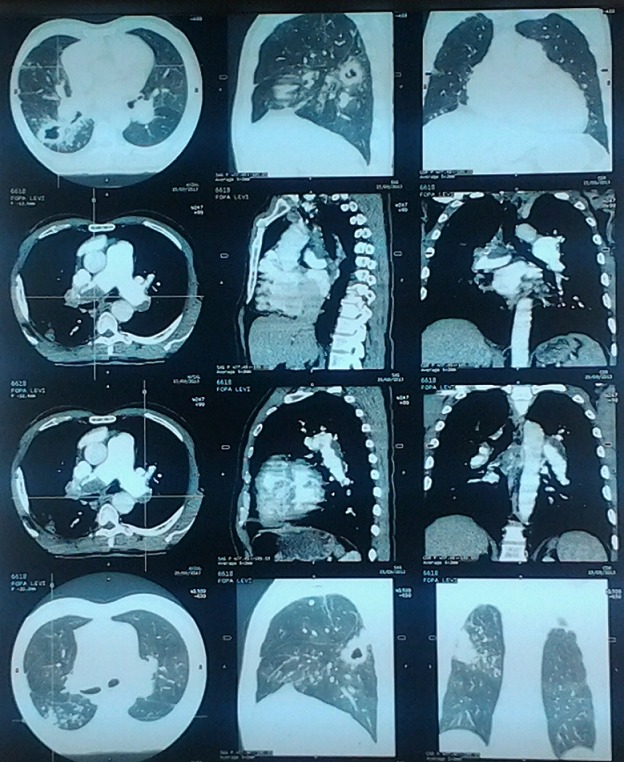
Thoracic computed tomographic scan showing pulmonary emboli and right lung cavitation

Chest radiograph revealed an enlarged cardiac silhouette, a right middle lung lobe cavitary lesion with regional lymphadenopathy. ECG showed normal sinus rhythm, incomplete right bundle branch block and T wave inversions in V1,V2 and V3. Echocardiography showed dilated right atrium, dilated right ventricle, severe tricuspid regurgitation and severe pulmonary artery hypertension; Pulmonary Artery Systolic Pressure (PASP): 80mmHg. Ejection fraction of the left ventricle was 70%. Following the results of the echocardiography, a thoracic Computed Tomographic scan with contrast was performed which showed bilateral proximal pulmonary emboli(Figure 1, Figure 2, Figure 3, Figure 4), and a cavitary lesion in the upper lobe of the right lung (Figure 2, Figure 3, Figure 4). The cavitation of the right lung prompted bronchial fibroscopy with aspiration of bronchial secretions. Sputum was found to contain numerous acid-fast bacilli on direct microscopy (>15 bacilli/field). Culture results was positive for mycobacterium tuberculosis. Brochial biopsy showed severe dysplasia of bronchial epithelium with absence of carcinoma. Ultrasound studies did not show evidence of deep venous thrombosis. Other laboratory findings revealed a WBC of 4300/mm^3^, hemoglobin 13.8 g/dL, platelet count 315,000/mm^3^, Serum creatinine 11.92mg/l, C-reactive protein 54.4mg/l, erythrocyte sedimentation rates at 46mm and 59mm,fasting blood sugar 1g/l, aspartate aminotransferase 19.9 IU/l, alanine aminotransferase 15.6IU/l, negative HIV serology, INR 1.10. He was put on enoxaparin that was overlapped with acenocoumarol. Anti-tuberculosis treatment including isoniazid, pyrazinamid, ethambutol and rifampicin was started immediately. He was discharged with referral to the pneumology hospital for continuation of treatment when the therapeutic INR was reached.

## Discussion

Tuberculosis (TB) is a major killer worldwide mainly affecting people of developing countries [1]. Our case shows bilateral pulmonary embolism associated with pulmonary tuberculosis, an association which has been reported previously [2, 3]. However, this complication of tuberculosis is very rare and the thrombogenic potential of tuberculosis has been infrequently reported in literature. However, patients with TB are predisposed to venous thromboembolism due to several pathophysiologic mechanisms, affecting all three parts of the Virchow's Triad [4–6]. Our patient did not have any risk factors for thromboembolism and had a low clinical probability for pulmonary embolism [7].

The first report of an association between tuberculosis and pulmonary embolism was in 1950, in which pulmonary embolism was found 27 times in 111 subjects of active tuberculosis (24.3%) from 634 autopsies compared to 23.1% incidence of pulmonary embolism in the entire series [3]. Other reports also demonstrate that thrombotic phenomena inpatients with pulmonary TB occur in other sites such as the lower extremity veins [2], hepatic veins [8] and cerebral venous sinuses [9]. The association between tuberculosis and inflammation, hemostatic changes and a hypercoagulable has been recently established [5, 10]. Deficiency of Antithrombin III, protein C andprotein S and elevated plasma fibrinogen levels,increased platelet aggregation seems to induce hypercoagulable state which improves with anti-tuberculosis treatment within four weeks [5]. Thus, treatment should be started early in association with anticoagulation. Moreover, oral anti coagulation has to be carefully monitored because of hepatic enzyme induction by anti-tuberculosis drugs which increases hepatic clearance of oral anticoagulant, resulting in higher doses of the drug. Our patient was ambulatory prior to admission. Absence of any prior morbidity and any family history of a coagulopathy suggests tuberculosis as the underlying cause of pulmonary embolism in the patient.

## Conclusion

This case report highlights that patients with pulmonary tuberculosis are at increased risk of thrombotic events; pulmonary embolism in the case of our patient. This is an entity that is rarely taken into consideration, which might pose a diagnostic dilemma and could play a major role in the outcome of the patient. Clinicians bearing this in mind, especially in developing country settings, could avert eventual thromboembolic events in Tuberculosis.

## References

[1] WHO (2004). The world health report 2004: changing history.

[2] Bishav Mohan, Anil Kashyap, Jagdeep Whig, Vineet Mahajan (2011). Pulmonary Embolism in cases of Pulmonary Tuberculosis: a unique entity. Indian J Tuberc..

[3] Morgan TJ (1950). Autopsy incidence of pulmonary embolism in tuberculosis. Chest..

[4] Robson SC, White NW, Aronson I, Woollgar R, Goodman H, Jacobs P (1996). Acute-phase response and the hypercoagulable state in pulmonary tuberculosis. Br J Haematol..

[5] Turken O, Kunter E, Solmazgul E, Cerrahoglu K, Ilvan A (2002). Hemostatic changes in active pulmonary tuberculosis. Int J Tuberc Lung Dis..

[6] Ambrosetti M, Ferrarese M, Codecasa LR, For the AIPO/SMIRA TB Study Group (2006). Incidence of venous thromboembolism in tuberculosis patients. Respiration..

[7] Wells PS, Anderson DR, Bormanis J (1997). Value of assessment of pretest probability of deep-vein thrombosis in clinical management. Lancet..

[8] Gogna A, Grover S, Arun A, Saluja S (2004). Isolated Hepatic InferiorVena Cava Thrombosis in a Case of Tuberculosis - Case Report. JIACM..

[9] Júnior JA, Felício A, Fukujima M, Rodrigues C, Morelli V, Lourenço D, Prado G (2005). An uncommon cause of cerebral venous thrombosis. ArqNeuropsiquiatr..

[10] Naithani R, Agrawal N, Choudhary VP (2007). Deep venous thrombosis associated with tuberculosis. Blood Coagul Fibrinolysis..

